# Operation of the Atypical Canonical Bone Morphogenetic Protein Signaling Pathway During Early Human Odontogenesis

**DOI:** 10.3389/fphys.2022.823275

**Published:** 2022-02-08

**Authors:** Xiaoxiao Hu, Chensheng Lin, Ningsheng Ruan, Zhen Huang, Yanding Zhang, Xuefeng Hu

**Affiliations:** Center for Biomedical Research of South China, Fujian Key Laboratory of Developmental and Neural Biology, College of Life Science, Fujian Normal University, Fuzhou, China

**Keywords:** tooth, development, Smad4-independent, atypical canonical BMP signaling, human dental mesenchymal cell

## Abstract

Bone morphogenetic protein (BMP) signaling plays essential roles in the regulation of early tooth development. It is well acknowledged that extracellular BMP ligands bind to the type I and type II transmembrane serine/threonine kinase receptor complexes to trigger the BMP signaling pathway. Then, the receptor-activated Smad1/5/8 in cytoplasm binds to Smad4, the central mediator of the canonical BMP signaling pathway, to form transfer complexes for entering the nucleus and regulating target gene expression. However, a recent study revealed the functional operation of a novel BMP-mediated signaling pathway named the atypical BMP canonical signaling pathway in mouse developing tooth, which is Smad1/5/8 dependent but Smad4 independent. In this study, we investigated whether this atypical BMP canonical signaling is conserved in human odontogenesis. We showed that pSMAD1/5/8 is required for the expression of Msh homeobox 1 (*MSX1*), a well-defined BMP signaling target gene, in human dental mesenchyme, but the typical BMP canonical signaling is in fact not operating in the early human developing tooth, as evidenced by the absence of pSMAD1/5/8-SMAD4 complexes in the dental mesenchyme and translocation of pSMAD1/5/8, and the expression of *MSX1* induced by BMP4 is mothers against decapentaplegic homolog 4 (SMAD4)-independent in human dental mesenchymal cells. Moreover, integrative analysis of RNA-Seq data sets comparing the transcriptome profiles of human dental mesenchymal cells with and without *SMAD4* knockdown by siRNA displays unchanged expression profiles of pSMAD1/5/8 downstream target genes, further affirming the functional operation of the atypical canonical BMP signaling pathway in a SMAD1/5/8-dependent but SMAD4-independent manner in the dental mesenchyme during early odontogenesis in humans.

## Introduction

The mouse tooth has long served as an excellent model system to study the molecular mechanism underlying mammalian odontogenesis. Bone morphogenetic protein (BMP) signaling has been demonstrated to be a fundamental player in mouse tooth development. The major BMP ligands, including *Bmp2*, *Bmp4*, and *Bmp7*, are found to be expressed in the epithelium and mesenchyme of the developing tooth germ in mice (Nie et al., 2006). Amid them, *Bmp4* is initially expressed in the dental epithelium at the laminar stage around E11 and subsequently induces the expression of *Msx1*, a well-known *Bmp4* downstream target gene, in the dental mesenchyme. At the following bud stage, the expression of *Bmp4* is shifted to the dental mesenchyme, which is activated by the mesenchymally expressed *Msx1* and is being maintained there until the late differentiation stage. This mesenchymal *Bmp4*, in turn, maintains *Msx1* expression by forming a positive regulatory loop with *Msx1*. Deletion of *Msx1* revealed a dramatic downregulation of Bmp4 in dental mesenchyme and exhibited an arrest of tooth development at the bud stage. Application of exogenous BMP4 to or ectopic expression of *Bmp4* in *Msx1* mutant dental mesenchyme can partially rescue tooth deficiency (Bei et al., 2000; Zhang et al., 2005). Meanwhile, mesenchymal BMP4 acts on the dental epithelium as a feedback signal to induce and maintain gene expression, such as *Shh* and *p21*, in the dental epithelium and is responsible for the formation of the enamel knot, a signaling center for tooth cusp patterning (Jernvall et al., 1998). In addition, *Bmp4*, together with *Bmp2* and *Bmp7*, is expressed in the enamel knot and responsible for apoptosis in the knot cell (Mitsiadis et al., 2010). Moreover, *Bmp4* also synergizes with *Msx1* to activate the mesenchymal odontogenic potential for sequential tooth formation by inhibiting the expression of *Dkk2* and *Osr2* (Jia et al., 2013). Taken together, BMP signaling is absolutely required for early tooth morphogenesis.

Activation of BMP signaling involves binding of BMP ligands to transmembrane type II and type I serine/threonine kinase receptors. Activated receptors transduce signals through canonical and non-canonical pathways (Nohe et al., 2004; Miyazono et al., 2010). In the canonical pathway, with binding of BMP ligands to receptors, the type II receptor phosphorylates the type I receptor and forms heterodimeric complexes, which in turn lead to phosphorylation of Smad1/5/8 (the receptor-regulated Smads, R-Smads) in the cytoplasm. pSmad1/5/8 then forms a complex with Smad4 (the common Smad, Co-Smad) and translocates to the nucleus to regulate target gene transcription (Nohe et al., 2004). In this currently accepted model, Smad4 has been regarded as the central mediator, playing an indispensable role for the nuclear translocation of pSmad1/5/8-Smad4 complex and the activation of downstream target gene expression during the signaling transduction (Lagna et al., 1996). However, previous studies also reported that the accumulation of Smad1/5 in the nucleus for the transduction of BMP signaling to trigger the expression of downstream target genes is independent of Smad4, and the deficiency in Smad4 causes no or mild defects in the development of several organs (Xu et al., 2008; Retting et al., 2009). Conditional knockout of *Smad4* in the dental mesenchyme does not reveal dental abnormality and alteration of *Msx1* expression in early tooth development (Li et al., 2011). These results obviously challenge the current model of the canonical BMP signaling pathway. Actually, a previous study in mice did demonstrate that this typical canonical signaling pathway is not, in fact, operating but a novel BMP signaling, named as the atypical canonical BMP signaling pathway, is functioning in developing mouse teeth, which is pSmad1/5/8-dependent but Smad4-independent (Yang et al., 2014).

Although the regulatory mechanism and function of BMP signaling have been studied and their importance has been established in the mouse model, it remains elusive whether this fundamental pathway is fully conserved or how it operated in humans. In this study, we aimed to further explore whether this atypical BMP/pSMAD1/5/8 canonical pathway is conserved in human tooth morphogenesis. We found that BMP-induced *MSX1* expression and pSMAD1/5/8 nuclear translocation is, in fact, SMAD4-independent in cap-stage human molar germs and human dental mesenchymal cells. In addition, unchanged expression profiles of genes downstream of pSMAD1/5/8 were confirmed by analysis of RNA-Seq data sets comparing the transcriptome profiles of human dental mesenchymal cells with and without *SMAD4* knockdown by siRNA. Our results demonstrate that the atypical canonical BMP signaling pathway is operating during early human odontogenesis.

## Materials and Methods

### Collection of Human Embryonic Tissues

Human embryonic jaws isolated from chemically aborted human fetuses of 12-week gestations were provided by the Maternal and Children Health Care Hospital of Fujian Province. Informed consent forms of utilizing aborted embryos for scientific research were approved by the participants. Experiments of the human embryonic tissues were performed following the stipulations of the Ethics Committee of Fujian Normal University.

### Organ Culture and Cell Culture

For organ culture, freshly separated human molar germs were cultured with the Trowell-type organ culture system in DMEM/10% fetal bovine serum (FBS) at 37°C and a 5% CO_2_ incubator. Isolation of primary human dental mesenchymal cells (hDMCs) and culture of immortalized human dental mesenchymal cells (ihDMCs) was carried out as previously described (Huang et al., 2015b). BMP4 protein (R&D Systems, 314-BP-050) was added to the medium at the final concentration of 100 ng/ml. For small-molecule inhibition experiments, dorsomorphin (Sigma, P5499), SB203580 (CST, 5633), U0126 (CST, 9903), and SP600125 (Abcam, ab120065) were added into the medium at the final concentration of 20 μM. Dimethyl sulfoxide (DMSO) (Sigma, 76314) was used as the negative control. Immunofluorescence and Western blotting were used to verify the efficiency after 24 h.

### RNA Interference, Immunocytochemical Staining, and Western Blotting

SMAD4 siRNA (Sigma, SASI_Hs01_00207794) or negative control siRNA were transfected into cells at a final concentration of 20 nM using Lipofectamine™ RNAiMAX Transfection Reagent (Thermo Fisher, 13778075) for 48 h. Transfections were performed when cells grow to 60–80% of the dishes. For immunofluorescence, tooth germs were fixed in 4% paraformaldehyde (PFA), embedded in paraffin, and sectioned at 6 μm for immunohistochemical staining. After blocking in 5% bovine serum albumin (BSA), tissue sections and cell slides were then incubated with primary antibodies at 4°C overnight. Secondary antibodies were incubated at room temperature for 1 h followed by DAPI (Life, D1306) staining (sections for 2 min and cell slides for 1 min). For Western blot, cells were lysed using RIPA with protease inhibitor (Roche, 30559) followed by sonication of five cycles at 4°C. Proteins were separated with 12% SDS-PAGE gel and transferred to the nitrocellulose membrane. Subsequently, the membrane was blocked in 5% non-fat powdered milk at room temperature and then incubated with primary antibodies at 4°C overnight. Secondary antibodies were incubated at room temperature for 1 h, followed by visualizing. Semiquantitative analysis of Western blot and immunofluorescence mean values was carried out using Image J.^1^

The following primary antibodies were used: anti-pSMAD1/5/9 (CST, 13820), anti-SMAD4 (Abcam, ab40759), anti-MSX1 (R&D Systems, AF5045), anti-pSMAD2/3 (Santa Cruz, sc11769), anti-ERK1/2 (Sigma, M5670), anti-JNK (Santa Cruz, sc-6254), anti-pP38 (CST, 4511), and anti-ACTIN (Santa Cruz, sc58673). Secondary antibodies included donkey anti-mouse IgG (H + L) highly cross-adsorbed secondary antibody, Alexa Fluor 488 (Thermo, A-21202), Alexa Fluor 488 donkey anti-goat Ig (H + L) (Life, A11055), Alexa Fluor 594 donkey anti-goat Ig (H + L) (Life, A11058), donkey anti-rabbit IgG, Alexa Fluor 488 (Thermo, A-21206), Alexa Fluor 680 donkey anti-rabbit IgG (H + L) (Thermo, A10043), Alexa Fluor 680 donkey anti-goat Ig (H + L) (Life, A21084), and Alexa Fluor 790 donkey anti-mouse IgG (H + L) (Life, A11371). All the experiments were performed according to the instructions of the manufacturer and repeated at least three times.

### *In situ* Proximity Ligation Assay

Tissue sections on glass slides were detected using Duolink^®^ *in situ* Red Starter Kit (Sigma-Aldrich, DUO92101). Slides were blocked with Duolink blocking solution in a preheated humidity chamber for 30 min at 37°C and then incubated with anti-pSMAD1/5/9 (CST, 9511), anti-pSMAD2/3 (Santa Cruz, sc11769), and anti-SMAD4 (Invitrogen, MA5-15682) antibodies in the same chamber overnight at 4°C. Samples were incubated with proximity ligation assay (PLA) probe solution (anti-mouse PLA probe Minus and anti-rabbit PLA probe Plus or anti-goat PLA Plus) for 1 h at 37°C and then incubated with a ligation solution for 30 min followed by amplification reaction for 100 min at 37°C. Slides were mounted with a coverslip using a minimal volume of mounting medium containing DAPI.

### RNA-Seq and Data Analysis

Total RNA of cultured hDMCs was extracted using the RNeasy Mini Kit (Qiagen, 74104). RNA samples were then used for quality control and library preparation. Illumilla HiSeq X Ten was used to perform sequencing using the 150-bp pair-end-read configuration. All the experiments were repeated three times. Data were analyzed using Galaxy. Reads were mapped to hg38 with HISAT2 (Kim et al., 2019) and were counted in genomic features using FeatureCounts software (Liao et al., 2014). DEseq2 was used to differ differential expressions (Love et al., 2014). TBtools was used to visualize Venn and for gene ontology (GO) enrichment analysis by default setting (Chen et al., 2020). Then, ggplot2 (Wickham, 2016) was used to visualize the GO enrichment result. The log_2_ transformed transcript level showed in the scatterplot was obtained using Seqmonk,^2^ and the scatterplot was visualized using MATLAB.^3^ The RNA-Seq data were deposited in the Gene Expression Omnibus (GEO) database (accession number: GSE179474).

## Results

### Presence of Bone Morphogenetic Protein Intracellular Signal Transducers During Early Human Odontogenesis

**FIGURE 1:**
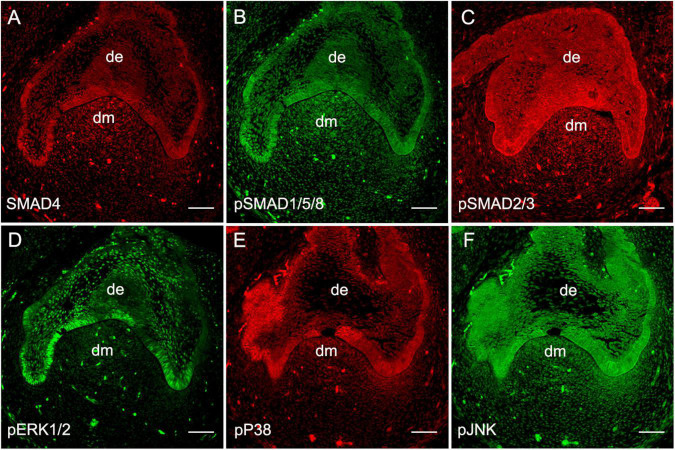
Expression of BMP intracellular signal transducers at the human cap-stage tooth germ. **(A–C)** Expression of SMAD signal transducers: SMAD4 **(A)**, pSMAD1/5/8 **(B)**, and pSMAD2/3 **(C)**. **(D–F)** Expression of MAPK signal transducers: pERK1/2 **(D)**, pP38 **(E)**, and pJNK **(F)**. de, the dental epithelium; dm, the dental mesenchyme. Bar = 50 μm.

To validate the functional operation of BMP signaling during human odontogenesis, based on our previous report on the expression patterns of BMP ligands, receptors, and antagonists in the human developing tooth germs (Dong et al., 2014), we first set out to further confirm the presence of BMP intracellular signal transducers during human tooth development using human cap-stage molar germs. Immunostaining showed that the major molecules that mediate canonical BMP signaling pathways including SMAD4 (Figure 1A) and pSMAD1/5/8 (Figure 1B) were abundantly present in the epithelium and mesenchyme of the tooth germs. We also detected an intense expression of SMAD2/3, the transducers involved in TGFβ signaling, that were overlapped with SMAD4 and pSMAD1/5/8 in the tooth germs (Figure 1C). Meanwhile, the expression of the central transducers of BMP/MAPK pathway pERK1/2, pP38, and pJNK was observed at a high level in the dental epithelium and mesenchyme, except for barely detectable pERK1/2 in the dental mesenchyme at this stage (Figures 1D–F). Thus, taken together with our previous report (Dong et al., 2014), all these observations indicate that BMP signaling, transduced both/either through SMADs and/or MAPK, is, in fact, operating during early human tooth development.

### Expression of *MSX1* Is Regulated by Bone Morphogenetic Protein/SMAD1/5/8 Signaling in Early Human Tooth Germs

**FIGURE 2:**
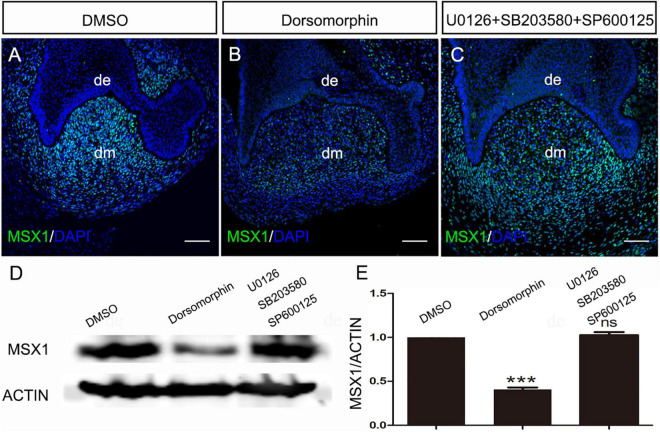
Regulation of MSX1 expression is mediated by pSMAD1/5/8 in the human cap-stage tooth germs. **(A–C)** Immunostaining shows that MSX1 expression is abundant in dimethyl sulfoxide (DMSO)-treated human cap-stage tooth germs, dramatically reduced in dorsomorphin treated human cap-stage tooth germ, and unaltered in U0126 + SB203580 + SP600125 treated human cap-stage tooth germs. **(D)** A Western blot assay confirms the dramatically reduced expression of MSX1 in dorsomorphin treated human cap-stage tooth germs. Actin was used as the internal control. **(E)** Quantitative analysis of the Western blot assay. de, the dental epithelium; dm, the dental mesenchyme. Error bars represent SD. ***p < 0.001. Bar = 50 μm.

*Msx1* has been demonstrated to be a BMP/Smad1/5/8 signaling target gene and is well known for its critical role in early tooth morphogenesis in mice (Jia et al., 2016). In humans, *MSX1* is also restricted to the dental mesenchyme as in mice (Lin et al., 2007) and is a crucial player in human odontogenesis as evidenced by the fact that mutation in *MSX1* results in Rieger syndrome, which exhibits severe tooth agenesis. To determine whether the regulation of *MSX1* expression by BMP/pSMAD1/5/8 is conserved during early human tooth development, we performed a small-molecule inhibition experiment using dorsomorphin (SMAD1/5/8 phosphorylation inhibitor) to specifically block transduction of BMP/pSMAD1/5/8 signaling pathway and combination of SB203508 (p38 MAPK inhibitor), U0126 (ERK1/2 inhibitor), and SP600125(JNK inhibitor) to specifically block BMP/MAPK signaling pathway (Xiao et al., 2017; Liu et al., 2018; Wang et al., 2019) in cap-stage human molar germs cultured *in vitro* with the Trowell-type organ culture system. Immunostaining showed that, after cultured for 24 h, the expression of *MSX1* was dramatically downregulated in dorsomorphin-treated molar tooth germs (Figure 2B), whereas it was hardly disturbed when treated with the combination of SB203508, U0126, and SP600125 (Figure 2C) as compared with that treated with DMSO (Figure 2A). This inhibition of *MSX1* expression by dorsomorphin but not SB203508, U0126, and SP600125 was further confirmed by Western blotting (Figure 2D), where more than 60% reduction in the *MSX1* expression level was quantified in dorsomorphin-treated human tooth germs compared with that in SB203508, U0126, and SP600125 co-treated human molar germs and DMSO-controls (Figure 2E). These results indicate that the regulation of *MSX1* expression is mediated by pSMAD1/5/8 signaling but not by MAPK in developing human dental mesenchyme.

### SMAD4 Is Not Required for Bone Morphogenetic Protein4/pSMAD1/5/8-Induced *MSX1* Expression in the Human Dental Mesenchymal Cells

**FIGURE 3:**
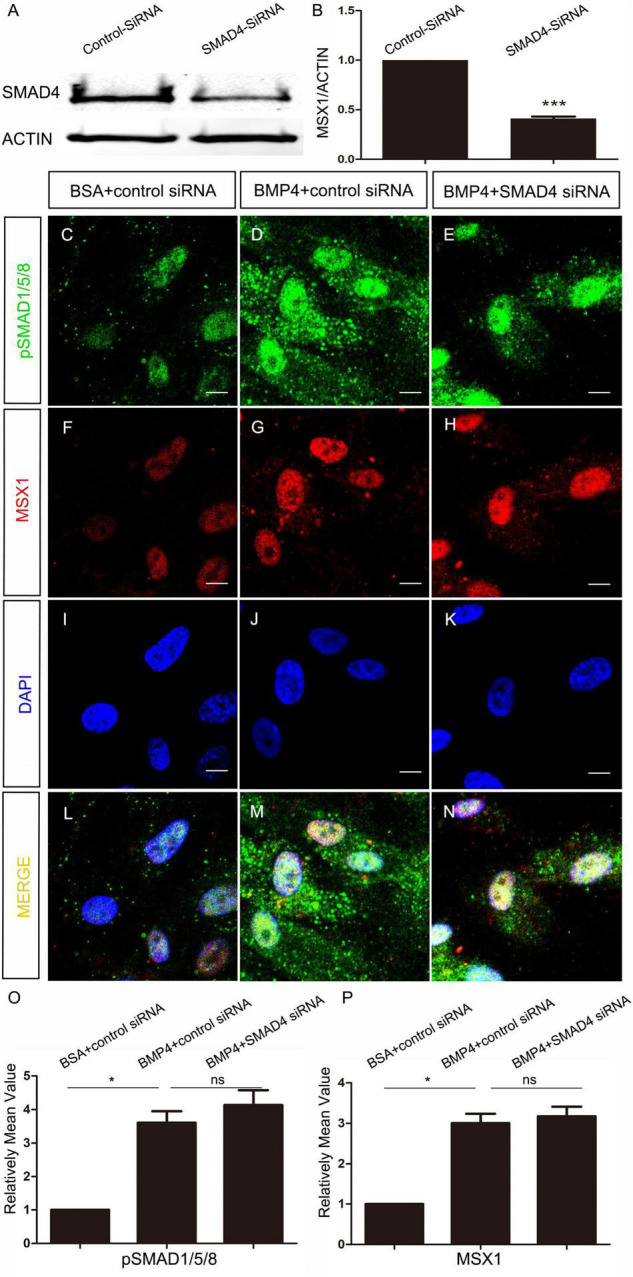
SMAD4 is not required for BMP4-induced pSMAD1/5/8 nuclear translocation and MSX1 expression in the hDMCs. **(A,B)** Western blot shows approximately 60% knockdown efficiency of SMAD4 siRNA. **(C–N)** Co-immunostaining of MSX1 and pSMAD1/5/8 show that BMP4-induced pSMAD1/5/8 nuclear translocation and MSX1 expression are not affected by knockdown of SMAD4. **(O,P)** Quantitative analysis of relative mean fluorescence values shows that pSMAD1/5/8 and MSX1 expressions are not affected by the knockdown of SMAD4. Error bars represent SD. ns, *p* > 0.05; *p < 0.05; and ***p < 0.001. Bar = 10 μm.

**FIGURE 4:**
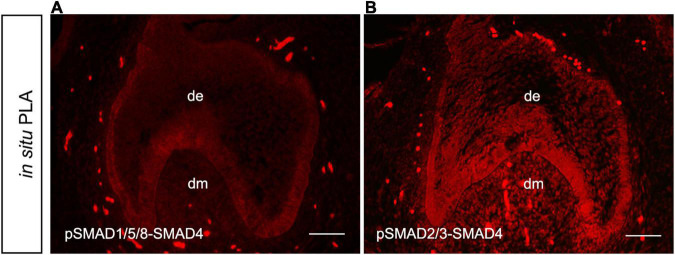
Absence of pSMAD1/5/8-SMAD4 complex in the hDMCs. *In situ* proximity ligation assay (PLA) shows barely detectable pSMAD1/5/8-SMAD4 complexes **(A)** but abundant pSMAD2/3-SMAD4 complexes **(B)** in hDMCs. Bar = 50 μm.

To elucidate whether *MSX1* expression regulated by BMP/pSMAD1/5/8 is also independent of SMAD4 in human dental mesenchyme as its mouse congener does, we then conducted SMAD4 siRNA knockdown experiments in hDMCs and ihDMCs that were isolated from bell-stage human molar germs and retains the expression of several tooth-specific markers including *MSX1* (Huang et al., 2015a). Approximately 60% knockdown efficiency of SMAD4 at the protein level was first verified by Western blotting in the hDMCs (Figures 3A,B) and ihDMCs (Supplementary Figures 1A,B) transfected with SMAD4 siRNA compared with that treated with control-siRNA for 48 h. Further RNAi experiments demonstrated that the nuclear translocation of pSMAD1/5/8 was SMAD4-independent as shown in Figures 3C–N and Supplementary Figures 1C–K. Weak nuclear staining of pSMAD1/5/8 was seen in the cultured hDMCs (Figures 3C,I,L) and ihDMCs (Supplementary Figures 1C,F,I), and this nuclear-located pSMAD1/5/8 became abundant after the addition of exogenous BMP4 and treated with control siRNA (Figures 3D,J,M and Supplementary Figures 1D,G,J), indicating that an active BMP-induced pSMAD1/5/8-translocation is operating in these cells. As expected, this nuclear translocation was not affected by *SMAD4* knockdown as evidenced by the presence of equally abundant nuclear pSMAD1/5/8 in these cultured cells when transfected with SMAD4 siRNA (Figures 3E,K,N and Supplementary Figures 1E,H,K) as compared with the control siRNA. Similarly, equally abundant MSX1 present in the nuclei of hDMCs (Figures 3F–N) and ihDMCs (Supplementary Figures 2A–I) treated with SMAD4 siRNA compared with that treated with control siRNA after induced with BMB4. The results were further confirmed by semiquantification (Figures 3O,P). Consistent with these findings, abundant pSMAD2/3-SMAD4 complexes (Figure 4A) but rare pSMAD1/5/8-SMAD4 complexes (Figure 4B), visualized by PLA [an assay for detecting protein-protein interaction with high specificity and sensitivity, (Renfrow et al., 2011)], were found in the human cap-stage molar germs, suggesting that pSMAD2/3 possesses higher binding affinity with SMAD4 than pSMAD1/5/8 in the context of developing human teeth, and the absence of pSMAD1/5/8-SMAD4 complex could be the consequence of saturated SMAD4 by SMAD2/3. Together, our results provide compelling evidence that SMAD4 is dispensable for *MSX1* expression regulated by BMP/pSMAD1/5/8 signaling during early human tooth development.

### Modulation of Bone Morphogenetic Protein/SMAD1/5/8 Signaling Cascade in a SMAD4-Independent Manner in the Human Dental Mesenchymal Cells

**FIGURE 5:**
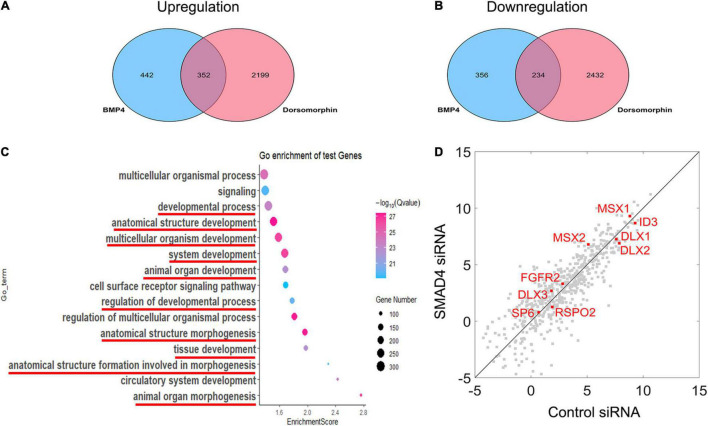
RNA-Seq analysis reveals that the modulation of BMP/pSMAD1/5/8 is SMAD4-independent. **(A,B)** Venn diagram showing the overlap between BMP4-induced and dorsomorphin-inhibited mRNA transcripts and the mRNA ≥1 × changed in hDMCs. **(C)** Gene ontology analysis shows that the upregulated and downregulated genes are primarily involved in development and morphogenesis (red line). The scatterplot reveals differences in transcript abundance between control siRNA-treated hDMCs and SMAD4 siRNA-treated hDMCs. **(D)** Transcript levels are log2 transformed. The genes that have been demonstrated to be crucial in tooth development and their regulatory domains (promoters) are directly bound by pSMAD1/5/8 as well are indicated in red.

Nuclearly translocated pSMAD1/5/8 not only activates *MSX1* expression but also triggers a signaling cascade in association with other transcription factors and transcriptional coactivators or corepressors. To identify the gene set that is involved in the BMP/pSMAD1/5/8 signaling cascade, we compared the difference in the genome-wide transcriptome among hDMCs treated with BSA, BMP4, and BMP4 plus dorsomorphin, respectively, using RNA-Seq. Comparing of RNA-Seq data between BSA- and BMP4-treated hDMCs revealed that 794 genes are upregulated and 590 genes downregulated by BMP4 proteins (Figures 5A,B, blue oval). As expression alteration of these genes was triggered by BMP proteins, they are considered to be both involved in BMP canonical and non-canonical pathways. To distinguish genes involved in BMP/pSMAD1/5/8 signaling cascade from genes belonging to the entire BMP signaling cascade, we further conducted RNA-Seq on the hDMCs treated with BMP4 + DMSO and BMP4 + dorsomorphin (pSMAD1/5/8 inhibitor), respectively. Comparison of these two RNA-Seq data sets identified 2,551 downregulated genes and 2,666 upregulated genes (Figures 5A,B, pink oval). Since dorsomorphin exerts an inhibitive effect on pSMAD1/5/8 function and would restrain its downstream target gene activity, the downregulated and upregulated genes identified in the above inhibitive experiment would be in an opposing situation, i.e., the downregulated would be the upregulated and vice versa, in the normal physiological condition in the hDMCs (Figures 5A,B). Comparison of genes associated with BMP signaling cascade and genes with pSMAD1/5/8 signaling cascade identified 586 overlapped genes that are involved in BMP/pSMAD1/5/8 signaling cascade with 352 genes upregulated and 234 genes downregulated when treated with BMP4 in hDMCs (Figures 5A,B and Supplementary Table 1). GO analysis showed that these genes are primarily involved in development and morphogenesis (Figure 5C). Several studies demonstrated that ∼60% RNAi efficiency of critical upstream genes was capable of altering the expression pattern of downstream genes by RNA-Seq analysis (Brooks et al., 2011; Sun et al., 2018; Smekalova et al., 2020). Therefore, we further performed RNA-Seq on the hDMCs treated with BMP4 + control siRNA and BMP4 + SMAD4 siRNA, respectively, to test if the expression levels of these 586 genes involved in the BMP/pSMAD1/5/8 signaling cascade are altered by knockdown of *SMAD4*. Comparison of RNA-Seq data from 352 upregulated genes and 234 downregulated genes is plotted in Figure 5D. The dots are distributed along the diagonal line although there are genes, particularly the genes with lower expression levels, apparently deviated from the diagonal line. These deviations could be the consequence of indirect regulation of them by pSMAD1/5/8 and crosstalk/interaction among the signaling pathways that constitute the complex signal network of the cell. Actually, when chasing down the genes that have been demonstrated to play crucial roles in tooth development and are directly bound by pSMAD1/5/8 at their promoter domains (Genander et al., 2014), including *MSX1*, *MSX2*, *SP6*, *FGFR2*, *ID3*, *DLX1*, *DLX2*, and *DLX3* (Zhang et al., 2003; Choi et al., 2010; Nakamura et al., 2012; Huang et al., 2015a; Aurrekoetxea et al., 2016; Gong et al., 2020; Hu et al., 2020; Figure 5D, red dot), we found that they are closely situated along the diagonal line, indicating that *SMAD4* is not required for modulation of pSMAD1/5/8 direct target genes when triggered with BMP4.

## Discussion

In this study, we provided compelling evidence that, as in the mouse, the atypical canonical BMP signaling pathway is in fact operating during early human tooth development. We demonstrated that, in the human dental mesenchyme, pSMAD1/5/8 are able to transduce BMP signal to regulate the expression of downstream target gene *MSX1* in a *SMAD4* independent manner, as evidenced by the fact that *MSX1* expression is inhibited by dorsomorphin in the human cap stage tooth germ and that knockdown of *SMAD4* by siRNA exhibits no effect on BMP-induced pSMAD1/5/8 nuclear translocation and *MSX1* expression in hDMCs. This notion is further strengthened by the fact that the BMP-induced expression of genes involved in BMP/SMAD1/5/8 signaling cascade, such as *MSX1*, *MSX2*, *SP6*, *FGFR2*, *RSPO2*, *ID3*, *DLX1*, *DLX2*, and *DLX3*, which have been demonstrated to play critical roles in tooth development and to their promoters pSMAD1/5/8 directly bind, is *SMAD4*-independent, as estimated by *SMAD4* knockdown and RNA-Seq analysis. PLA shows that the cytoplasmic SMAD4 in human dental mesenchymal cells is saturated by pSMAD2/3 to form pSMAD2/3-SMAD4 complexes, a central signal transduction element for TGFβ/Smad2/3 signaling pathways, which also play important functions in tooth development (Ohta et al., 2018).

Since the partnership between SMAD4 and mothers against decapentaplegic homolog (SMAD) proteins in TGFβ signaling pathways was verified in *Xenopus* embryos and breast epithelial cells (Lagna et al., 1996), numerous studies have demonstrated that *SMAD4* functioning as a central mediator (common-partner SMAD) is indispensable in BMP/SMAD1/5/8 and TGFβ/activin/2/3 signaling pathways that exert essential functions during embryonic development and are also involved tissue homeostasis and regeneration in the adults (Nickel and Mueller, 2019). However, this widely accepted model that Smad4 is indispensable for SMAD signal transduction has been challenged by *Smad4* knockout studies in several developing organs. Specific inactivation of Smad4 in the early mouse epiblast resulted in a profound failure to pattern derivatives of the anterior primitive streak, including prechordal plate, node, notochord, and definitive endoderm, whereas the TGFβ- and BMP-regulated processes involved in mesoderm formation and patterning are unaffected, which results in the normal formation of the allantois, a rudimentary heart, somite, and lateral plate mesoderm. These results suggest that *Smad4* is dispensable for some tissue and organ formation during early embryonic development (Chu et al., 2004). Conditional knockout of *Smad1* and *Smad5* introduced severe phenotypes including cerebellar hypoplasia, reduced granule cell numbers, and disorganized Purkinje neuron migration, whereas conditional inactivation of *Smad4* resulted in only very mild cerebellar defects during nervous system development (Tong and Kwan, 2013). *Smad4*-independent events were also found in TGFβ signaling pathways. For instance, TGFβ/Smad2/3-dependent Mad1 induction and keratinocyte differentiation are independent of *Smad4* during cell-cycle exit and differentiation of suprabasal epidermal keratinocytes. It is IκB kinase but not Smad4 serves as a nuclear cofactor for Smad2/3 recruitment to *Mad1* chromatin (Descargues et al., 2008). Yang et al. (2014) demonstrated that the BMP canonical signaling pathway is operating in a Smad1/5/8-dependent but *Smad4*-independent manner in the dental mesenchyme during marine early odontogenesis, named as the atypical BMP canonical signaling pathway. The absence of pSmad1/5/8-Smad4 complexes is the consequence of the saturation of Smad4 by Smad2/3 in the dental mesenchymal cells. In this investigation, we provided compelling evidence that this atypical BMP canonical signaling pathway is fully conserved in humans.

According to the study by Nickel and Mueller, the dilemma for the SMAD study is that many growth factors but just two principal signaling pathways: “A hallmark of the TGF protein family is that all of the more than 30 growth factors identified to date signal by binding and hetero-oligomerization of a very limited set of transmembrane serine-threonine kinase receptors. […] This discrepancy indicates that our current view of TGF signaling initiation just by hetero-oligomerization of two receptor subtypes and transduction *via* two main pathways in an on-off switch manner is too simplified” (Nickel and Mueller, 2019). The report by Yang et al. (2014) together with this study implies that tinkering of the SMAD signaling by weeding out extant components, such as SMAD4, and adding additional components, such as IκB kinase (Descargues et al., 2008), may allow diversification of signal transduction and downstream target gene activation at the cellular level. This certainly warrants further attention.

Although the human and mouse teeth share considerable homology throughout the developmental stages (Zhang et al., 2005), they do manifest heterodont dentition and apparently various morphologies including developmental phase, shape, and pattern. It is suggested that the diversity of tooth morphology resulted from the tinkering of the conserved signal pathway instead of creating novel ones during evolution (Tummers and Thesleff, 2009). Our recent studies revealed distinct expression patterns of genes involved in the major conserved signal pathways, such as SHH (Hu et al., 2013), WNT(Wang et al., 2014), FGF(Huang et al., 2015a), and BMP (Dong et al., 2014) signaling. For instance, our previous study showed that, as in the mouse, BMP ligands including BMP2, 3, 4, and 7 are also expressed in the cap and bell stages of human tooth germs (Dong et al., 2014). However, these genes are expressed in a broad and persistent pattern in both the dental epithelium and mesenchyme throughout the early cap to the late bell stage in humans, unlike in mice their expression is restricted to the limited region of the tooth germ, such as enamel knot, and appears in limited periods of time. We believe that this spatial-temporal difference in gene expression would confer engaged cells and/or tissues with specific biological behavior such as proliferation, apoptosis, and differentiation and result in characteristic tooth morphology of their own. In this study, we found that the central intracellular signal transducer involved in the BMP/MAPK signaling pathway, such as pERK1/2, pP38, and pJNK, and involved in SMAD signaling, including SMAD4, SMAD1/5/8, and SMAD2/3, are all present in the cap-stage human tooth germ, indicating operating and importance of BMP signaling during early human odontogenesis. We demonstrated that an intracellular BMP signal transduction pathway, the atypical canonical BMP signaling pathway, is fully conserved between humans and mice. Our results provide evidence that the diversity of tooth morphology in different species may be resulted from tinkering of the extracellular signal molecules with distinct distributing pattern instead of tinkering with intracellular signal transduction in mammal.

A large portion of the human population has congenitally missing teeth, and the probability of tooth loss increases with the age of a person. The pressing demand for replacement teeth in regenerative dental medicine has brought up a matter of great urgency to explore the molecular mechanisms that regulate tooth development in humans. However, the major bulk of our current knowledge on tooth development derives from studies on mice. Unveiling the molecular basis involved in human tooth morphogenesis will provide important insight for studying genetically related dental abnormalities and tooth regeneration in humans.

## Data Availability Statement

The datasets presented in this study can be found in online repositories. The names of the repository/repositories and accession number(s) can be found in the article/Supplementary Material.

## Ethics Statement

The studies involving human participants were reviewed and approved by Ethics Committee of Fujian Normal University. The patients/participants provided their written informed consent to participate in this study.

## Author Contributions

XXH contributed to the conception, experimental data acquisition and analysis, and draft. CL and NR contributed to experimental data acquisition. ZH contributed to data analysis. XFH and YZ contributed to conception and manuscript revision. All authors contributed to the article and approved the submitted version.

## Conflict of Interest

The authors declare that the research was conducted in the absence of any commercial or financial relationships that could be construed as a potential conflict of interest.

## Publisher’s Note

All claims expressed in this article are solely those of the authors and do not necessarily represent those of their affiliated organizations, or those of the publisher, the editors and the reviewers. Any product that may be evaluated in this article, or claim that may be made by its manufacturer, is not guaranteed or endorsed by the publisher.

## Footnotes

1
https://imagej.nih.gov/ij/index.html

2
www.bioinformatics.babraham.ac.uk/projects/seqmonk/

3
www.mathworks.com/products/matlab.html
