# Supplementary material for: Operation of the Atypical Canonical Bone Morphogenetic Protein Signaling Pathway During Early Human Odontogenesis
Source: Front Physiol. 2022 Feb 8;13:823275. doi: 10.3389/fphys.2022.823275 (PMC8863179; doi:10.3389/fphys.2022.823275)
Supplement: Supplementary file 2 [file Data_Sheet_1.PDF]

Operation of the atypical canonical BMP signaling pathway during early human odontogenesis

Supplementary material

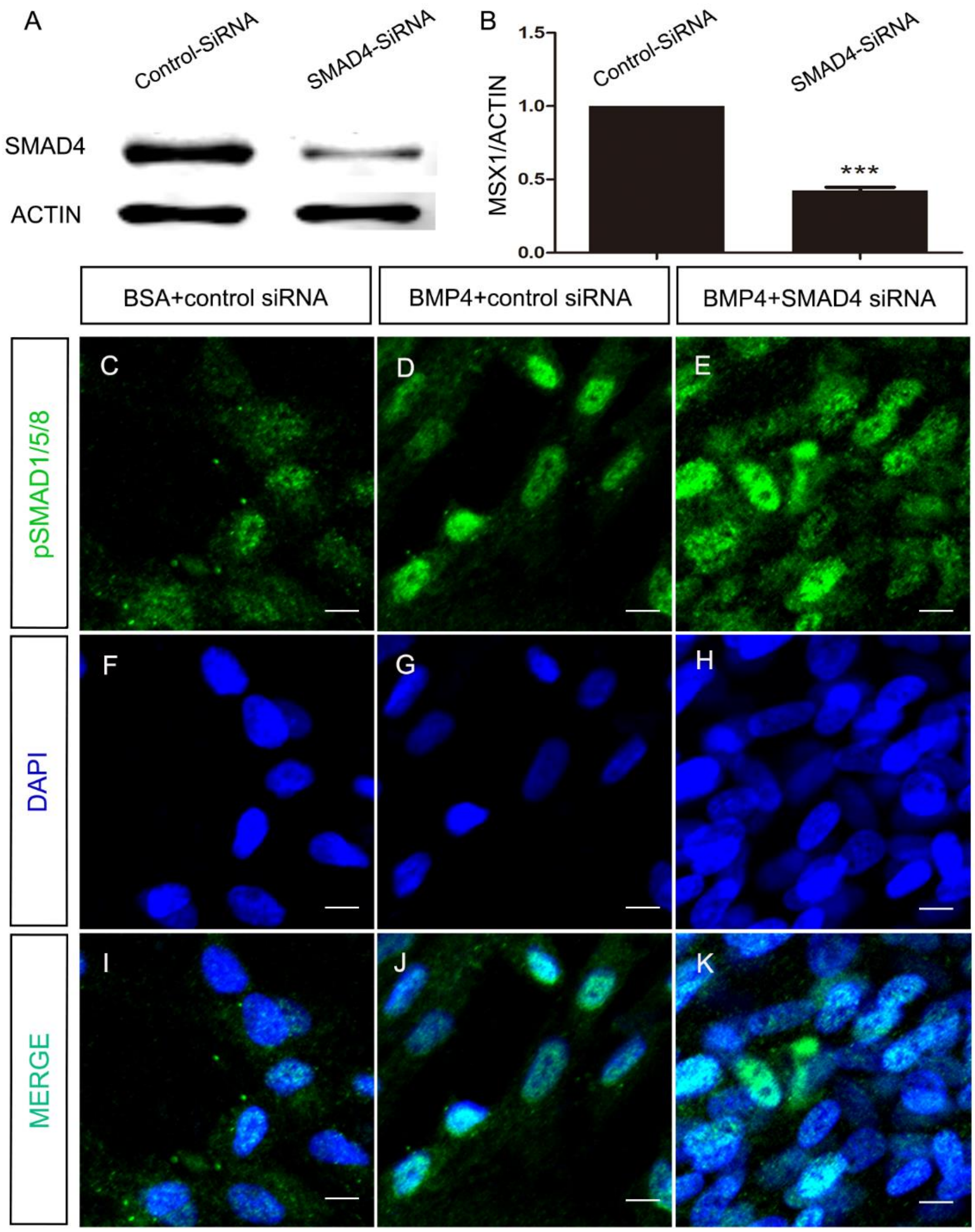

Supplementary Figure 1. SMAD4 is not required for BMP4-induced pSMAD1/5/8 nuclear translocation in the ihDMCs. (A, B) Western blot shows about 60% knockdown efficiency of SMAD4 siRNA. **(C-K)** Immunostaining shows that BMP4-induced nuclear translocation of pSMAD1/5/8 is not effected by knockdown of *SMAD4*. Error bars represent standard deviation. ***, $p < 0.001$. Bar=10um.

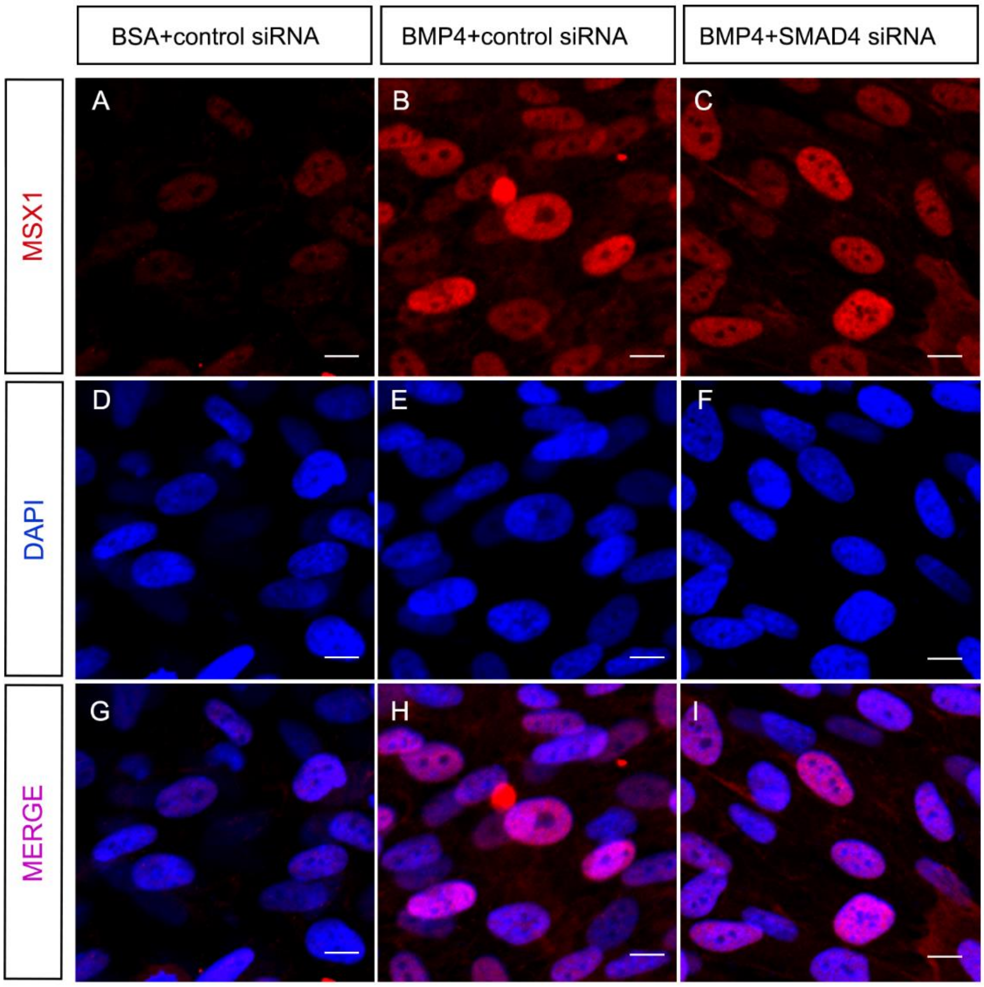

Supplementary Figure 2. SMAD4 is not required for BMP4-induced Msx1 expression in the ihDMCs. (A-I) Immunostaining shows that BMP4-induced MSX1 expression is not effected by knockdown of *SMAD4*. Bar=10um
